# Factors Influencing the Control of Diabetes Measured via Glycated Hemoglobin Concentrations in Adults with Type 1 Diabetes

**DOI:** 10.3390/ejihpe13100144

**Published:** 2023-09-24

**Authors:** Beata I. Sińska, Ewa Rzońca, Alicja Kucharska, Robert Gałązkowski, Iwona Traczyk, Patryk Rzońca

**Affiliations:** 1 Department of Human Nutrition, Faculty of Health Sciences, Medical University of Warsaw, 01-445 Warsaw, Poland; alicja.kucharska@wum.edu.pl (A.K.); iwona.traczyk@wum.edu.pl (I.T.); 2 Department of Obstetrics and Gynecology Didactics, Faculty of Health Sciences, Medical University of Warsaw, 00-575 Warsaw, Poland; erzonca@wum.edu.pl; 3 Department of Emergency Medical Services, Faculty of Health Sciences, Medical University of Warsaw, 00-575 Warsaw, Poland; robert.galazkowski@wum.edu.pl; 4 Department of Human Anatomy, Faculty of Health Sciences, Medical University of Warsaw, 02-004 Warsaw, Poland; patryk.rzonca@wum.edu.pl

**Keywords:** type 1 diabetes, glycated hemoglobin, adult, predisposing factor

## Abstract

Numerous complications of type 1 diabetes (T1D) may be prevented through suitable glycemic control. Glycated hemoglobin (HbA1c) may be one of the markers for the early detection of the metabolic imbalance characteristic of the disease. However, optimal control of diabetes is not achieved in a large group of patients. It was demonstrated that numerous factors (sociodemographic, psychological, and clinical) contributed to this condition. The aim of the study was to identify factors influencing the control of diabetes measured via glycated hemoglobin concentrations in people with T1D. Independent factors influencing better diabetes control measured via HbA1c in the study group included higher disease acceptance, higher nutritional adherence, lower BMI, and a lower risk of eating disorders. Describing the determinants will allow for the improvement of the system of care provided to people with T1D and for it to comprise important psychological variables related to self-care and acceptance of the disease.

## 1. Introduction

Diabetes is one of the most widely distributed chronic diseases and is often portrayed as a silent disease [1,2]. Type 1 diabetes (T1D) is characterized by the destruction of insulin-producing β-cells in the pancreas. Insulin deficiency leads to hyperglycemia, which requires the administration of exogenous insulin to prevent life-threatening acute and chronic complications [3]. Patients with type 1 diabetes are at an increased risk of cardiovascular disease, polyneuropathy, nephropathy, and retinopathy, which may shorten life expectancy in people with uncontrolled diabetes [4]. However, this risk may be reduced through intensive glycemic control, aiming to achieve optimal glycated hemoglobin (HbA1c) levels. Measuring HbA1c is a standard and preferred test by which to assess long-term glycemic control and possibly predict the risk of diabetes complications. With prolonged hyperglycemia, a large amount of hemoglobin is glycated, which consequently reflects the metabolic control of diabetes and represents the average glucose levels over the past 8–12 weeks [5]. Any decrease in HbA1c levels (through strict glycemic control) significantly reduces the rate of the progression of microvascular complications. Conversely, any increase in HbA1c may raise the incidence of cardiovascular events by up to 18% and the incidence of microvascular complications by up to 30% [6].

In people with diabetes, the overall goal of glycemic control, in terms of HbA1c value, according to the recommendations of the Polish Diabetes Association (2023) [7], is not to exceed 7.0% (53 mmol/mol), which corresponds to an average daily glycemia of 154 mg/dl (in the range of 123–185 mg/dl). Obviously, the goal of diabetes therapy is to control daily glucose levels and achieve adequately low HbA1c levels while optimizing the patient’s quality of life.

Despite significant technological and pharmacological advances in the treatment of diabetes, fewer than one-third of patients with T1D achieve optimal metabolic control [8].

It was demonstrated that numerous factors contributed to poor glycemic control, including older age, female sex, alcohol consumption, higher body mass index (BMI), smoking, longer duration of the disease, lower physical activity, failure to follow therapeutic recommendations and many others [9].

Studies indicated that glycemic control [10] and the process of the metabolic control of diabetes were largely influenced by psychological variables related to the degree of involvement in self-care and acceptance of the disease. As regards following dietary recommendations, it is especially important for the patient to feel responsible for their health, to accept the disease, and to be motivated. Psychosocial, environmental, and social factors also influence the life and medical therapy of T1D patients [11]. Failure to include all interfering factors in the educational and therapeutic programs used in T1D therapy may contribute to their poor effectiveness [12].

Recognizing and understanding the factors that determine good metabolic control of diabetes is crucial for better management of type 1 diabetes. Therefore, the aim of the research was to analyze the factors affecting the control of type 1 diabetes mellitus measured via the concentration of glycated hemoglobin.

## 2. Materials and Methods

### 2.1. Participants and Study Design

The observational cross-sectional online questionnaire study was conducted on 417 people with type 1 diabetes between November 2021 and January 2023. Nonprobability sampling of study participants was used. The inclusion criteria were as follows: age 18 and more, at least 1-year history of type 1 diabetes, and informed consent to participate in the study. The study was anonymous, and participation was voluntary. The questionnaire was distributed online or individually to groups of patients with type 1 diabetes and personal contacts of the members of the study group (i.e., the snowball method).

A questionnaire consisting of two parts was used as the research tool. The present authors’ part consisted of collecting data on the sociodemographic aspects of the respondents and clinical information related to diabetes (duration of the disease, number of episodes of hypo- and hyperglycemia, number of insulin units per kg of body weight). The second part used the following:

- Diabetes Dietary Guidelines Adherence Index (the DDGA Index): This index combines current recommendations concerning healthy eating for the population and the guidelines of behavioral therapy of the Polish Diabetes Association. The index takes account of the regular consumption of meals and the recommended frequency of the consumption of 29 groups of products. One point was scored if the frequency of the consumption of a specific group of products adhered to the recommendations, while 0 was scored in case of non-adherence. The DDGA Index value was expressed as the total score between 0 and 30 points. Higher DDGA Index values were interpreted as a higher degree of adherence to dietary recommendations (i.e., 0 points—complete lack of adherence to the recommendations; 30 points—complete adherence to the recommendations) [13].
- The Acceptance of Illness Scale (AIS) as adapted by Jurczyński: This scale contains 8 statements describing the consequences of poor health regarding the recognition of limitations imposed by the disease, lack of self-sufficiency, a sense of dependence on other people, and lowered self-esteem. The answers were scored from “strongly agree”−1 to “strongly disagree”−5. The total, ranging from 8 to 40, is a general measure of the degree of acceptance of the disease, with low scores indicating poor adaptation to the disease, while high scores indicate acceptance of the disease. The greater the acceptance, the better the adaptation and the lower the sense of psychological discomfort [14].
- The Diabetes Eating Problem Survey-Revised scale (DEPS-R): A diabetes-specific tool for screening eating disorders. The DEPS-R consists of 16 items, each containing 6 responses on a 6-point Likert scale. The overall DEPS-R score ranges from 0 to 80, so those with higher total DEPS-R scores are more likely to have an eating disorder. According to the original version of the DEPS-R, the total score equal to or above 20 was set as a threshold point indicating greater disturbances [15].
- The Sense of Responsibility for Health Scale (HSRS), developed by Adamus: The scale consists of 12 items rated on a 5-point scale (1—hardly ever, 2—rarely, 3—sometimes, 4—often, 5—nearly always/very often). The HSRS allows for the determination of the total level of the sense of responsibility for one’s health (HSRS-T) and includes two subscale scores: Active Involvement (HSRS-AI) and Adequate Behaviour (HSRS-AB). Only the total level of responsibility for one’s health was assessed in the present study. This is due to the fact that the HSRS-AI and HSRS-AB subscores are correlated [16].

### 2.2. Ethics

Due to the anonymous character of the questionnaire and the impossibility of following up on sensitive data, the study required no approval of the Bioethics Committee. However, the authors sought some advice from the Bioethics Committee of the Medical University of Warsaw in conducting the present study. As the “committee does not issue opinions on the survey, respective and other non-invasive scientific studies”, no approval was required. Data owners gave their permission to use the data.

### 2.3. Statistical Analysis

The collected data were subjected to statistical analysis using the STATISTICA software, version 13.2 (Tibco Software Inc., Palo Alto, CA, USA). Group statistics are presented as numbers (n) and percentages (%) for categorical variables and M ± SD for continuous variables, where M is mean and SD is standard deviation. As explained later for the tables, n(%) is too easily confused with M(SD). Normal distribution of variables was tested with the Kolmogorov–Smirnov and Lilliefors tests. The comparison of categorical variables was made using the Chi^2^ test for categorical variables, and the comparison of continuous variables was performed with the Mann–Whitney U test. In correlation analysis, the Spearman correlation coefficient was used. The assessment of the impact of selected factors predisposing a normal glycated hemoglobin result was made on the basis of single logistic regression analysis, where each factor was considered separately. Then, multivariable logistic regression analysis was performed, which included all relevant predictors predisposing the normal HbA1c result. The odds ratio (OR) and 95% confidence intervals (95%CI) were used to measure the strength of the relationship between the dependent variable and the predictors. The statistical significance threshold was set at *p* < 0.05.

## 3. Results

Data were collected from 417 T1D patients, of whom 189 had HbA1c levels at or below 7% and 228 had HbA1c levels over 7%. Those with HbA1c ≤ 7% were more often found in women (50.2% vs. 39.0%), people living in urban areas (48.3% vs. 35.7%), those declaring tertiary education (53.4% vs. 40% or less), and those with lower BMI (24.3 ± 4.0 vs. 26.2 ± 4.3) (Table 1). The above-mentioned associations were statistically significant (*p* < 0.05). No significant association was noted between HbA1c levels and age (*p* > 0.05). Detailed data are presented in Table 1.

Moreover, HbA1c results below 7% were more likely to be found in patients with type 1 diabetes with higher disease acceptance (M = 28.4), higher adherence to dietary recommendations based on the DDGA Index (M = 18.9), lower risk of developing eating disorders in patients with type 1 diabetes based on the DEPS-R (M = 13.2), and a higher sense of responsibility for one’s health (M = 57.7). Study participants with glycated hemoglobin results below 7% were characterized by a lower daily number of insulin units per kilogram of body weight (M = 0.54), the absence of episodes of hyperglycemia (53.3%) or their occurrence 1–2 times a week (56.1%), and knowing the calorie value of their diet (51.3%). The above-mentioned associations were statistically significant (*p* < 0.05). No significant association was noted between HbA1c levels and disease duration, type of insulin therapy, and hypoglycemic episodes in subjects with type 1 diabetes (*p* > 0.05) (Table 2).

**Figure 1:**
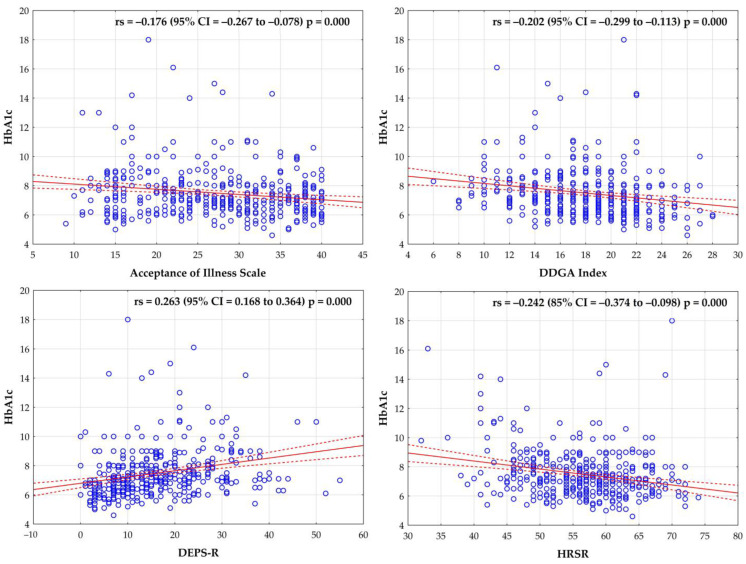
Correlation between the level of glycated hemoglobin and the Acceptance of Illness Scale, DDGA Index, DEPS-R Scale, and HRSR.

Statistical analysis showed significant weak negative correlation between the level of glycated hemoglobin and the Acceptance of Illness Scale (r = −0.174), DDGA Index (r = −0.195), and HRSR (r = −0.240), and a weak positive correlation between the level of glycated hemoglobin and DEPS-R (r = 0.264) (*p* < 0.05) (Figure 1).

**Figure 2:**
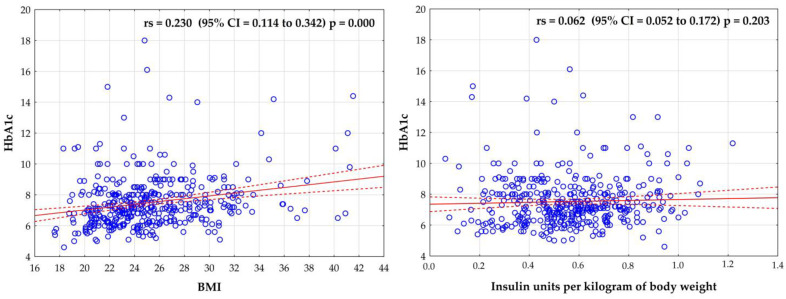
Correlation between the level of glycated hemoglobin and BMI and insulin units per kilogram of body weight per day.

The statistical analysis also showed a statistically significant weak positive correlation between glycated hemoglobin and BMI (r = 0.230; *p* < 0.05). No correlation was noted between HbA1c levels and daily number of insulin units per kilogram of body weight (*p* > 0.05) (Figure 2).

Table 3 presents the analysis of the logistic regression of factors predisposing a normal glycated hemoglobin result. The analysis of univariate logistic regression showed ten factors related to the maintenance of a normal glycated hemoglobin result, i.e., female sex, living in an urban area, tertiary education, BMI, disease acceptance level, degree of adherence to dietary recommendations, the risk of eating disorders, the level of the sense of responsibility for one’s health, knowledge of the daily number of insulin units, and knowing the calorie value of one’s diet (*p* < 0.05). Conversely, the multivariate logistic regression analysis showed that a higher level of disease acceptance (OR = 1.03; 95%CI 1.00–1.06; *p* < 0.05) and higher degrees of adherence to dietary recommendations (OR = 1.08; 95%CI 1.02–1.14; *p* < 0.05) were associated with a higher frequency of better hemoglobin level control below 7% in the examined patients. In turn, a lower BMI (OR = 0.92; 95%CI 0.87–0.97; *p* < 0.05) and a lower risk of eating disorders (OR = 0.96; 95%CI 0.94–0.99; *p* < 0.05) contributed to a better control of diabetes, i.e., below 7% HbA1c in subjects with type 1 diabetes.

## 4. Discussion

The identification of factors influencing glycemic control in patients with T1D is essential for the effective treatment of this disease and is of interest to many researchers. So far, studies have most often described factors such as sociodemographic factors (young age, low level of education), adherence to dietary recommendations, methods of insulin administration, and monitoring blood glucose [17,18,19]. In addition to the above-mentioned factors, the present study also drew attention to psychosocial factors: the acceptance of the disease, a sense of responsibility for one’s health, and the risk of eating disorders. Seemingly, a multidimensional approach may be helpful in developing individualized educational programs with higher effectiveness.

The results obtained in the present study indicated sex as an important determinant of the discussed issue—women had lower HbA1c concentrations than men. Relationships between glycemic control described in the literature are not entirely consistent. Similarly to the present study, Xing et al. (2022) reported that the prevalence of good glycemic control was lower in men than in women [20]. In turn, the results of Duarte et al. (2019) showed that women with T2D had poorer glycemic control than men [21]. A similar relationship was indicated in a systematic review and meta-analysis by Bitew et al. (2023), who stated that being a man protected against poor glycemic control [22]. It may be assumed that the differences in glycemic control between sexes may be due to differences in the regulation of glucose homeostasis, individual responses to treatment, and psychological factors [21].

Numerous scientists emphasized inequalities in the health of people with diabetes in the context of their place of residence, with poorer health results being associated with inhabiting rural areas [23,24,25,26]. A study on the trends in diabetes-related mortality in urban and rural areas in the USA between 1999 and 2019 revealed a temporary decrease in diabetes-related mortality in cities and among women [23]. The results of our research also demonstrated that urban residence was a factor affecting the control of diabetes and was associated with maintaining normal glycated hemoglobin results below 7%.

Level of education is another factor associated with poorer glycemic control and a higher risk of complications [3,27,28,29]. The present study showed that having a tertiary education was associated with maintaining a normal glycated hemoglobin result below 7% in subjects with type 1 diabetes. Similar observations in the context of education and diabetes were presented in a paper by Allen and McFarland (2020), showing that education and income provided important social and economic resources, the consequences of which varied in terms of the level of HbA1c depending on whether type 2 diabetes was diagnosed or not. Social resources resulting from the level of education may be more important in delaying the occurrence of the disease, while economic resources provided by income may be more important during the treatment of the disease [28].

Univariate logistic regression analysis also showed that the BMI index was an important factor related to maintaining a normal glycated hemoglobin level; its lower value was related to better control of diabetes. Dahlström et al. (2019) reported that normal body weight was optimal for people with type 1 diabetes and might translate into a reduced risk of death, especially in men and people in whom diabetes was diagnosed later in life [30].

Another variable analyzed in the paper was the level of disease acceptance. The result obtained in the present study indicates the average acceptance of the disease by the respondents (28.4 points). The results are consistent with those of other authors. In a study by Badura-Brzoza et al. (2022), the level of disease acceptance was 29.7 points in patients with type 1 diabetes and 31.6 points in those with type 2 diabetes [31]. Bonikowska et al. (2021) demonstrated that the level of AIS was 28.5 points in elderly patients with type 2 diabetes [32]. The analysis of the literature on the subject showed that the occurrence of diabetes complications, such as pain in patients with complicated diabetic foot syndrome after lower limb amputation [33] or poor hand function in patients with diabetes [34], contributed to lower acceptance of the disease. A study by Schmitt et al. showed that people with low disease acceptance were four times more likely to have HbA1c values above 9.0%, twice as likely to be diagnosed with long-term complications, and more than twice as likely to have had episodes of severe hypoglycemia and ketoacidosis over the past year [35]. It should be emphasized that a high level of acceptance of the disease means adaptation to the disease and its negative consequences. Moreover, people who accept the disease understand it better, are more positive about it, and are ready to actively participate in the entire therapeutic process and cooperate with healthcare professionals [36,37]. The results of our study showed that higher disease acceptance resulted in a better control of diabetes measured via HbA1c below 7%.

Alongside insulin administration, following the recommendations of a healthy diet is a key aspect of therapy for patients with T1D. Adherence to these recommendations correlated with insulin therapy provides satisfactory glycemic control [7], which reduces the risk factors for T1D complications, including cardiovascular complications and better weight control. High nutritional awareness of T1D patients is a factor conducive to a higher quality diet [38]. Furthermore, the results of the study confirmed that adherence to dietary recommendations prepared by a certified dietitian might be associated with a 1.0–1.9% decrease in HbA1c in people with T1D [39] and body weight within normal limits (BMI< 25 kg/m^2^) [19]. The present study demonstrated that patients with better-controlled diabetes (HbA1c below 7%) were characterized by a higher degree of adherence to dietary recommendations based on the DDGA Index (M = 18.9 points). In addition, their body mass index was within normal limits.

Eating disorders are a common problem in patients with T1D [40]. Data from Europe and North America using the DEPS-R questionnaire indicated incidence of such disorders at the level of 15–40% in people with T1D [41]. The risk of eating disorders, weaker glycemic control, and higher BMI increased with higher DEPS-R scores (>20 points) [42,43]. The results of our study showed a lower risk of developing eating disorders among people with lower HbA1c concentrations and lower BMI (M = 13.2 points). These results are consistent with other studies in which the DEPS-R score was lower in the group of T1D patients adhering to a diet compared to those who did not [18,44], and with those in which poorer metabolic control in T1D was identified along with a higher risk of eating disorders [41,45].

The study showed that psychological scales such as the DDGA, AIS, DEPS-R, and HSRS indices showed significant relevance not only in single analyses, as they also seemed to play a key role in the logistic regression model. Including variables that define the psychological characteristics and attitudes of patients may be a promising direction in creating personalized educational programs or other forms of support with which to improve the self-management of the disease and the quality of life in people with diabetes.

The conducted study analyzed numerous psychological, sociodemographic, and clinical factors in the context of the metabolic control of diabetes measured via glycated hemoglobin. However, there are some limitations. First, all the data obtained come from the online survey and are based on the statements of the respondents. The researchers did not verify whether the declarations of the respondents regarding their dietary behaviors were true; the principle of confidence was implemented in this study. Second, data on glycated hemoglobin concentration were also declared by people with T1D. Another limitation is related to the procedure of selecting patients for the study, as they were not selected randomly. Despite these limitations, our study is one of the few conducted in our country on a group of adult patients with T1D; previous studies were conducted in people with T2D or in groups of children and adolescents with T1D. The results of our study may contribute to a better understanding of the relationship between glycated hemoglobin concentrations and a number of factors in adults with T1D associated with being at a higher risk of poor metabolic control of the disease.

## 5. Conclusions

The independent factors influencing better diabetes control measured via HbA1c in a study group of patients with T1D included higher disease acceptance, higher nutritional adherence, lower BMI, and a lower risk of eating disorders based on the DEPS-R.

The results of our study highlighted the factors influencing the control of diabetes measured via glycated hemoglobin in subjects with type 1 diabetes. The identified determinants and the differences between them are necessary to improving the care provided to people with type 1 diabetes. Moreover, they emphasize the importance of psychological care, which is necessary in order to cover patients from the moment of their diabetes diagnosis.

## Figures and Tables

**Table 1:** Association of levels of glycated hemoglobin with sociodemographic factors.

Variables	HbA1c ≤ 7% (n = 189)	HbA1c > 7% (n = 228)	Total (n = 417)	*p*-Value
Age—*n* (%)				
19–30 years	89 (43.0)	118 (57.0)	207 (49.6)	0.615
31–50 years	85 (48.0)	92 (51.9)	177 (42.5)	
>50 years	15 (45.5)	18 (54.6)	33 (7.91)	
Gender—*n* (%)				
Female	118 (50.2)	117 (49.8)	235 (56.4)	0.023
Male	71 (39.0)	111 (60.9)	182 (43.6)	
Place of residence—*n* (%)				
Rural area	35 (35.7)	63 (64.3)	98 (23.5)	0.029
Urban area	154 (48.3)	165 (51.7)	319 (76.5)	
Education—*n* (%)				
Primary/vocational	9 (24.3)	28 (75.7)	37 (8.8)	0.001
Secondary	69 (40.1)	103 (59.9)	172 (41.3)	
Tertiary	111 (53.4)	97 (46.6)	208 (49.9)	
BMI—M ± SD	24.3 ± 3.9	26.2 ± 4.3	25.3 ± 4.3	<0.001

HbA1c—glycated hemoglobin; BMI—body mass index.

**Table 2:** Association of levels of glycated hemoglobin with psychological and clinical factors.

Variables	HbA1c ≤ 7% (n = 189)	HbA1c > 7% (n = 228)	Total (n = 417)	*p*-Value
Acceptance of Illness Scale—M ± (SD)	28.4 ± 8.2	26.1 ± 8.5	27.2 ± 8.4	0.004
DDGA Index—M ± (SD)	18.9 ± 4.2	17.14 ± 3.9	17.9 ± 4.1	<0.001
DEPS-R Scale—M ± (SD)	13.2 ± 10.3	19.01 ± 9.7	16.4 ± 10.4	<0.001
HRSR—M ± (SD)	57.7 ± 6.8	55.18 ± 7.8	56.3 ± 7.5	0.001
Insulin units per kilogram of body weight per day—M ± (SD)	0.54 ± 0.2	0.58 ± 0.2	0.56 ± 0.2	0.046
Duration of diabetes (years)—M ± (SD)	18.7 ± 9.50	18.8 ± 8.8	18.8 ± 9.1	0.629
Type of insulin therapy—n (%)				
Pens	98 (44.8)	121 (55.3)	219 (52.5)	0.804
Insulin pumps	91 (45.9)	107 (54.0)	198 (47.5)	
Hypoglycemic episodes—n (%)				
Every day	12 (57.1)	9 (42.9)	21 (5.04)	0.268
3 and more times a week	85 (41.7)	119 (58.3)	204 (48.9)	
1–2 times a week	62 (50.8)	60 (49.2)	122 (29.3)	
Never	30 (42.9)	40 (57.1)	70 (16.8)	
Hyperglycemic episodes—n (%)				
Every day	22 (29.7)	52 (70.3)	74 (17.8)	0.002
3 and more times a week	74 (42.3)	101 (57.7)	175 (41.9)	
1–2 times a week	69 (56.1)	54 (43.9)	123 (29.5)	
Never	24 (53.3)	21 (46.7)	45 (10.8)	
Knowing the calorie value of one’s diet—n (%)				
Yes	98 (51.3)	93 (48.7)	191 (45.8)	0.024
No	91 (40.3)	135 (59.7)	226 (54.2)	

HbA1c—glycated hemoglobin; M—mean; SD—standard deviation; DDGA—Index—Diabetes Dietary Guidelines Adherence Index; DEPS-R—Diabetes Eating Problem Survey-Revised Version Scale; HSRS—Sense of Responsibility for Health Scale.

**Table 3:** Analysis of the logistic regression of factors predisposing a normal glycated hemoglobin result.

	Univariate Logistic Regression			Multivariate Logistic Regression		
Variable	Orc	95% CI	*p*-Value	Ora	95% CI	*p*-Value
Sex (Female)	1.58	1.07–2.34	0.023	-	-	-
Place of residence (Urban area)	1.68	1.05–2.68	0.030	-	-	-
Education (Tertiary)	1.37	1.13–1.66	0.001	-	-	-
BMI	0.89	0.84–0.94	<0.001	0.92	0.87–0.97	0.004
Acceptance of Illness Scale	1.49	1.14–1.96	0.004	1.03	1.00–1.06	0.026
DDGA Index	1.11	1.06–1.17	<0.001	1.08	1.02–1.14	0.008
DEPS-R	0.94	0.92–0.96	<0.001	0.96	0.94–0.99	0.001
HSRS	1.05	1.02–1.08	0.001	-	-	-
Insulin units per kg BW	0.29	0.12–0.74	0.009	-	-	-
Hyperglycemic episodes	0.85	0.68–1.05	0.132	-	-	-
Knowing the calorie value of one’s diet	1.56	1.05–2.31	0.024	-	-	-

Orc—crude odds ratio; Ora—adjusted odds ratio; 95% CI—95% confidence interval; BMI—body mass index; DDGA Index—Diabetes Dietary Guidelines Adherence Index; DEPS-R—Diabetes Eating Problem Survey-Revised Version Scale; HSRS—Sense of Responsibility for Health Scale; BW—body weight.

## Data Availability

The data presented in our study are available on request from the corresponding author.
